# The mediating role of psychological resilience in the relationship between health literacy and health promotion behaviors among older adult patients undergoing PCI in China

**DOI:** 10.3389/fpubh.2025.1734318

**Published:** 2026-01-08

**Authors:** Yuxin Li, Tianxia Zhao, Ping Dai, Yanhong Wen, Yuting Fan, Jijun Wu, Lin He

**Affiliations:** 1 Department of Nursing, Deyang People’s Hospital, Deyang, Sichuan, China; 2 Department of Respiratory and Critical Care, Deyang People’s Hospital, Deyang, Sichuan, China; 3 Department of Cardiology, Deyang People’s Hospital, Deyang, Sichuan, China

**Keywords:** health literacy, health promotion behaviors, mediating effect, older adult, percutaneous coronary intervention, psychological resilience

## Abstract

**Objective:**

This study investigates whether psychological resilience mediates the relationship between health literacy and health promotion behaviors among older adult patients after PCI. The findings provide theoretical support for targeted interventions aimed at improving health promotion behaviors in this group.

**Methods:**

This cross-sectional study employed convenience sampling to survey 299 older adult patients who underwent PCI in the cardiovascular department of a Grade A tertiary hospital in Sichuan Province, China, from March to July 2024. Data collection employed questionnaires to gather general information, as well as the health promotion lifestyle scale, the health literacy scale, and the psychological resilience scale. Data analysis was conducted using SPSS 26.0 software for descriptive analysis, univariate analysis, correlation analysis, and multivariate stratified regression analysis. Mediating effect analysis was conducted using Model 4 in the Process v4.1 plugin.

**Results:**

The mean health promotion behavior score among older adult PCI patients was (82.13 ± 11.78). Correlation analysis revealed positive correlations between health literacy and health promotion behaviors (*r* = 0.840, *p* < 0.01), between health literacy and psychological resilience (*r* = 0.844, *p* < 0.01), and between psychological resilience and health promotion behaviors (*r* = 0.811, *p* < 0.01). Mediation analysis revealed that psychological resilience mediated the relationship between health literacy and health promotion behaviors, with an effect size of 0.260. This mediation accounted for 34.0% of the total effect (BootSE = 0.043，95%CI = [0.175, 0.343]).

**Conclusion:**

Health promotion behaviors among older adult patients after PCI were at a moderate level, and psychological resilience mediated the relationship between health literacy and health promotion behaviors. This suggests that healthcare providers should prioritize enhancing health literacy and psychological resilience among older adult PCI patients to improve their health promotion behaviors.

## 1 Introduction

Coronary heart disease (CHD), one of the leading cardiovascular diseases causing death and disability worldwide, has seen continuously rising incidence and mortality rates, becoming a major public health issue (1). Survey data indicate that as of 2019, approximately 197 million people globally suffered from CHD, with the prevalence projected to increase by 18.0% by 2030 (2). China currently has 11.39 million people living with CHD, and projections indicate that over 3.4 million deaths in China will be attributable to CHD by 2035 (3). Particularly noteworthy is that with the acceleration of population aging, the older adult population—often burdened by multiple chronic diseases, declining physiological functions, and reduced psychological resilience—has become the primary cohort affected by CHD, facing more complex clinical conditions and heavier health burdens (4). Percutaneous coronary intervention (PCI), a vital revascularization technique for treating CHD, uses cardiac catheterization to reopen narrowed or blocked coronary arteries. This effectively improves myocardial blood flow and rapidly alleviates symptoms (5). In recent years, with continuous advancements in interventional technology, PCI has become increasingly widespread in China, particularly as a key reperfusion therapy for older adult CHD patients. Reports indicate that by 2022, the number of PCI patients in China had reached 1.421 million, accounting for 23.2% of all hospitalized CHD patients (6). However, as a palliative revascularization therapy, PCI effectively improves coronary blood flow but cannot reverse the pathological progression of coronary atherosclerosis nor eliminate the fundamental risk factors of coronary heart disease (7). Therefore, patients who undergo PCI still require long-term health promotion behaviors to control risk factors and prevent recurrent cardiovascular events.

Health promotion behaviors refer to a series of proactive actions individuals take to maintain and enhance their own health, primarily encompassing six areas: nutrition, physical activity, health responsibility, interpersonal relationships, spiritual growth, and stress management (8). Research confirms that sound health promotion behaviors not only help delay disease progression, reduce readmission rates and mortality, but also significantly enhance patients’ quality of life and mental health (9). However, existing studies indicate that post-PCI older adult patients face suboptimal overall health promotion behaviors due to multiple factors, including age-related cognitive decline, limited information access channels, insufficient social support, and polypharmacy (10, 11). Furthermore, while PCI effectively addresses vascular stenosis, its long-term efficacy is highly dependent on patients’ post-procedural health-promoting behaviors, such as adhering to medication regimens, maintaining a balanced diet, engaging in regular exercise, and attending scheduled follow-up appointments. Extensive research reveals that older adult PCI patients in China commonly exhibit significant issues, including insufficient physical activity, poor dietary habits, and low medication adherence (12). These behaviors not only directly increase the risks of restenosis and readmission while driving up healthcare costs, but also severely compromise patients’ quality of life and long-term prognosis. Therefore, in-depth exploration of the factors influencing health promotion behaviors among older adult PCI patients and their underlying mechanisms holds significant practical importance for developing targeted intervention strategies and improving patient clinical outcomes.

Health literacy, defined as an individual’s ability to access, understand, and apply health information to make sound health decisions, serves as a critical precursor influencing health-promoting behaviors (13). Multiple studies indicate that the health literacy level of patients with coronary heart disease is significantly associated with their self-management behaviors and health outcomes. Patients with higher health literacy levels demonstrate greater understanding of disease-related knowledge, actively engage in disease management, and establish and maintain healthy lifestyles, thereby improving disease outcomes (14, 15). However, previous surveys of patients following PCI procedures indicate that this group generally exhibits low health literacy levels. They face significant difficulties particularly in understanding medication instructions, adhering to follow-up guidance, and recognizing acute symptoms. These challenges may directly impede their ability to effectively implement rehabilitation plans, thereby compromising postoperative recovery outcomes (16, 17). Research confirms that health literacy, a critical cognitive resource, positively predicts self-management capabilities and health outcomes in patients with CHD (18). Furthermore, prior studies indicate a positive correlation between health literacy levels and health-promoting behaviors among older adults. When older adults possess higher health literacy, they demonstrate greater adherence to rehabilitation guidance and are more likely to develop and sustain beneficial health-promoting behaviors, such as regular medication use, balanced diets, and scheduled follow-up appointments (19).

With the rise of positive psychology, psychological resilience has gradually emerged as a key psychological resource variable in health behavior research. Psychological resilience refers to an individual’s ability to quickly recover and adapt in the face of adversity, trauma, or stress (20). Research indicates that patients with higher psychological resilience tend to adopt more positive attitudes toward illness, strengthen treatment confidence, and improve adherence to self-management behaviors, thereby fostering stable health-promoting actions (21). For older adult individuals who have undergone major cardiac events (PCI), the procedure itself, the threat of disease, and functional limitations often impose significant psychological stress and adaptation challenges. This can erode their psychological resilience, thereby affecting their motivation and ability to actively engage in rehabilitation (12). Furthermore, studies demonstrate that psychological resilience, as a positive psychological resource, can regulate individuals’ cognitive processing of health information. When patients possess higher health literacy, their psychological resilience further facilitates the internalization and application of health information, ultimately enhancing the implementation of health-promoting behaviors (22).

**Figure 1:**
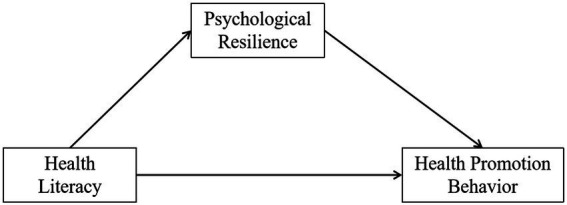
Research hypothesis mediation model.

The Health Promotion Model was first proposed by American nurse Nancy Pender in 1982. Integrating nursing and behavioral medicine, the model draws upon Expected Value Theory and Social Cognitive Theory to form a conceptual framework (23). It identifies factors influencing health promotion behaviors and is now widely applied in the management of chronic diseases, geriatric care, and related fields. The model identifies three determinants of health promotion behaviors: personal characteristics and experiences, behavior-specific cognitions and affections, and behavioral outcomes. It emphasizes that personal characteristics and experiences can directly influence behavioral outcomes or indirectly affect them through behavior-specific cognitions and affections. Health literacy refers to the relatively stable cognitive abilities and knowledge reserves that individuals develop through long-term learning and life experiences. It directly influences how individuals perceive and process health information, forming the cognitive foundation for health behavior decisions. It can be regarded as an important personal trait and experience (13). Psychological resilience refers to the positive psychological regulation processes individuals mobilize and exhibit when confronting specific health threats or recovery pressures. It involves cognitive assessments of stress, emotional responses, and adaptive strategies. This process directly influences an individual’s motivation, confidence, and persistence in adopting health-promoting behaviors, falling within the behavioral-specific cognitive and emotional domain (20). Currently, while limited research suggests associations between health literacy, psychological resilience, and health behaviors, direct empirical evidence remains scarce regarding the intrinsic mechanisms linking these three factors—particularly whether psychological resilience plays a key mediating role in the influence of health literacy on health-promoting behaviors—among the vulnerable population of older adult Chinese patients post-PCI. Therefore, grounded in the Health Promotion Model and addressing the documented deficiencies in health behaviors and health literacy among the aforementioned Chinese older adult PCI patients, alongside the potential pivotal role of psychological resilience, this study proposes the following research hypothesis (see Figure 1): Health literacy, as both a personal trait and experiential construct, not only directly predicts health promotion behaviors (behavioral outcomes) but may also indirectly foster the initiation and maintenance of such behaviors by influencing psychological resilience (behavior-specific cognition and emotion) when individuals confront rehabilitation challenges. This study aims to provide theoretical foundations for healthcare professionals to develop targeted interventions improving health promotion behaviors among older adult post-PCI patients and further advance healthy aging.

## 2 Methods

### 2.1 Study design

This study is a cross-sectional study.

### 2.2 Participants

Using convenience sampling, older adult patients undergoing PCI procedures in the Cardiovascular Department of a Grade A tertiary hospital in Sichuan Province, China, were selected as study subjects from March to July 2024. Inclusion criteria: ① Patients meeting diagnostic and treatment criteria for coronary heart disease, fulfilling indications for PCI, and in stable physical condition post-PCI (24); ② Patients aged ≥60 years; ③ Patients with clear consciousness capable of verbal or written communication; ④ Patients who provided informed consent and voluntarily participated in the study. Exclusion criteria: ① Patients with cognitive or psychiatric disorders, or severe visual/hearing impairments; ② Patients with severe concomitant systemic diseases (e.g., liver, brain, and kidney disorders), tumor recurrence/metastasis, or severe infectious diseases. Sample size was calculated using G*Power software (version 3.1) (25). Based on Cohen’s standards (26), the power was set at 0.95, the alpha level at 0.05, and the effect size *f*^2^ at 0.15 (considered a medium effect size). The study involved 23 independent variables. Using these parameters, G*Power calculated a required sample size of 234. Accounting for a 10% non-response rate, the sample size was adjusted to 260. Ultimately, 299 patients were enrolled, meeting the aforementioned criteria.

### 2.3 Instruments

#### 2.3.1 General information questionnaire

A self-designed general information questionnaire was administered, covering gender, age, marital status, number of children, education level, place of residence, medical payment methods, average monthly family income, number of chronic diseases, disease duration, number of interventional treatments, number of stent implants.

#### 2.3.2 Health literacy scale

The health literacy scale was developed by Jordan et al. (27) and adapted into Chinese by Sun (28). It comprises four dimensions—information acquisition ability, communication and interaction ability, willingness to improve health, and willingness to seek financial support—totaling 24 items. It employs a 5-point Likert scale, yielding a total score ranging from 24 to 120 points. Responses from “very difficult” to “not difficult at all” are scored from 1 to 5 points, respectively. Higher scores indicate greater health literacy. This scale has been widely applied among chronic disease patients and demonstrates good reliability and validity, with a Cronbach’s *α* coefficient of 0.890 (28). In this study, the Cronbach’s α coefficient for the scale was 0.943.

#### 2.3.3 Psychological resilience scale

The psychological resilience scale was developed by Campbell-Sills and Stein (20) and adapted into Chinese by Wang et al. (29). This scale comprises 10 items. It employs a 5-point Likert scale ranging from “Never” to “Always,” scored from 0 to 4 points, respectively. The total score ranges from 0 to 100 points, with higher scores indicating greater psychological resilience. This scale has been widely applied among chronic disease patients and demonstrates good reliability and validity, with a Cronbach’s *α* coefficient of 0.910 (29). In this study, the Cronbach’s α coefficient for this scale was 0.856.

#### 2.3.4 Health promotion lifestyle scale

The health promotion lifestyle scale was developed by American scholars Walker et al. (30) and adapted into Chinese by Cao et al. (31). This scale comprises six dimensions—nutrition, physical activity, health responsibility, interpersonal relationships, spiritual growth, and stress management—totaling 40 items. It employs a 4-point Likert scale, yielding a total score ranging from 40 to 160 points. Responses from “Never” to “Always” are scored as 1 to 4 points, respectively, with higher scores indicating better health-promoting behaviors. This scale has been widely applied among chronic disease patients and demonstrates good reliability and validity, with a Cronbach’s *α* coefficient of 0.930 (31). In this study, the Cronbach’s α coefficient for the scale was 0.941.

### 2.4 Data collection

This study employed a paper-based questionnaire survey method for data collection. Prior to the formal survey, researchers provided standardized training to three survey administrators, covering questionnaire completion procedures and key considerations. Following training, research subjects were rigorously selected based on inclusion and exclusion criteria. Prior to the survey, standardized instructions were used to thoroughly explain the study’s purpose, significance, questionnaire completion methods, and precautions to participants. Paper questionnaires were distributed only after obtaining patient consent and after the patient had signed the informed consent form. For subjects unable to complete the questionnaire independently, researchers assisted by reading each item aloud to them. Disease-specific information was completed by researchers after reviewing electronic medical records or inquiring with patients. Throughout the questionnaire completion process, researchers avoided prompting or interfering with patients. Completed questionnaires were collected immediately by researchers, who promptly reviewed them for completeness. Any omissions were addressed by requesting participants to supplement the information. A total of 315 questionnaires were distributed. After excluding 16 questionnaires with logical errors or patterned responses, 299 valid questionnaires were recovered, yielding a valid recovery rate of 93.4%.

### 2.5 Data analysis

Statistical analysis was performed using SPSS 26.0. Normally distributed continuous data were described using mean ± standard deviation, while categorical data were described using frequency and proportion. Intergroup comparisons were conducted using independent t-tests or one-way ANOVA. Pearson correlation analysis was employed to explore relationships between variables. Multivariate stratified regression analysis was employed to investigate the factors influencing health promotion behaviors among older adult PCI patients and to examine the mediating effects of these variables. The mediating effect of psychological resilience on the relationship between health literacy and health promotion behaviors was analyzed using Model 4 in the Process v4.1 plugin. Bootstrap testing was performed with 5,000 samples and a 95% confidence interval (CI). Effects were considered significant when the 95% CI did not include zero. The two-tailed significance level was *α* = 0.05.

### 2.6 Ethical considerations

The study followed the Declaration of Helsinki and was approved by the Ethics Committee of Deyang People’s Hospital (2024–04-018-K01). All study subjects gave informed consent and voluntarily participated in this study.

## 3 Results

### 3.1 General characteristics of older adult patients after PCI

Among the 299 older adult patients who underwent PCI in this study, 177 (59.2%) were male and 122 (40.8%) were female. The majority were aged 70 to <80 years (49.2%), and most were married (73.2%). Additional general characteristics are presented in Table 1.

**Table 1:** General characteristics of older adult patients after PCI and their relationship with health promotion behaviors.

Items	*N*(%)	Mean ± SD	*t/F*	*P*
Gender			−0.284	0.776
Male	177(59.2)	81.97 ± 11.80		
Female	122(40.8)	82.36 ± 11.79		
Age (years)			14.822	<0.001
60 ~ <70	128(42.8)	86.23 ± 9.39		
70 ~ <80	147(49.2)	79.06 ± 12.60		
≥80	24(8.0)	79.04 ± 11.80		
Marital status			6.487	<0.001
Married	219(73.2)	84.63 ± 10.88		
Single/Divorced/Widowed	80(26.8)	75.28 ± 11.47		
Number of children			0.621	0.538
1	144(48.2)	82.08 ± 11.74		
2 ~ 3	144(48.2)	82.47 ± 11.95		
≥4	11(3.7)	78.36 ± 10.26		
Education level				
Elementary and below	186(62.2)	78.50 ± 10.42	29.680	<0.001
Junior	63(21.1)	86.37 ± 10.11		
High school and above	50(16.7)	90.28 ± 12.79		
Place of residence			6.191	<0.001
Towns	134(44.8)	86.54 ± 11.96		
Countryside	165(55.2)	78.95 ± 10.35		
Medical payment methods			17.340	<0.001
Urban medical insurance	112(37.5)	87.00 ± 12.03		
Rural medical insurance	118(39.5)	79.71 ± 9.73		
Self-funded	69(23.1)	78.35 ± 12.03		
Average monthly family income (yuan)			52.747	<0.001
<3,000	46(15.4)	74.28 ± 10.52		
3,000 ~ <5,000	190(63.5)	80.42 ± 10.49		
≥5,000	63(21.1)	93.02 ± 8.68		
Number of chronic diseases			24.809	<0.001
1	57(19.1)	87.93 ± 10.47		
2	115(38.5)	84.79 ± 10.38		
≥3	127(42.5)	77.11 ± 11.60		
Disease duration (years)			47.464	<0.001
<5	78(26.1)	89.14 ± 10.47		
5 ~ <10	95(31.8)	85.04 ± 9.61		
≥10	126(42.1)	75.59 ± 10.65		
Number of interventional treatments			27.789	<0.001
1	155(51.8)	86.01 ± 10.99		
2	121(40.5)	78.52 ± 11.20		
≥3	23(7.7)	74.96 ± 10.79		
Number of stent implants			19.692	<0.001
1	56(18.7)	87.79 ± 11.52		
2	98(32.8)	84.84 ± 10.10		
≥3	145(48.5)	78.11 ± 11.57		

### 3.2 Univariate analysis of health promotion behaviors among older adult patients after PCI

Univariate analysis revealed statistically significant differences (*p* < 0.05) in health promotion behavior scores among older adult patients after PCI across various demographic factors: age, marital status, education level, place of residence, medical payment methods, average monthly family income, number of chronic diseases, disease duration, number of interventional treatments, and number of stent implant (see Table 1).

### 3.3 Common method bias test

To further enhance the rigor of this study, the Harman single-factor analysis method was employed to conduct an unrotated exploratory factor analysis on all scale items. The results revealed 15 factors with eigenvalues greater than 1 without rotation. The first factor explained 33.74% of the variance, falling below the 40% critical threshold. Therefore, it can be concluded that no significant common method bias exists in this study.

### 3.4 Descriptive analysis and correlation analysis of each scale

In this study, older adult patients who underwent PCI achieved a health literacy score of 69.02 ± 10.25, a psychological resilience score of 24.51 ± 3.98, and a health promotion behavior score of 82.13 ± 11.78. Correlation analysis revealed positive correlations among health literacy, psychological resilience, and health promotion behavior (*p* < 0.01) (see Table 2).

**Table 2:** Descriptive and correlation analysis of health literacy, psychological resilience, and health promotion behaviors.

Variables	Mean ± SD	1	2	3
1 Health literacy	69.02 ± 10.25	1		
2 Psychological resilience	24.51 ± 3.98	0.844*	1	
3 Health promotion behaviors	82.13 ± 11.78	0.840*	0.811*	1

^*^P < 0.01.

### 3.5 Multivariate stratified regression analysis

In the first step of the multivariate stratified regression, variables significant in the univariate analysis of health promotion behaviors were added to the model as control variables. After controlling for these variables in the second step, health literacy was found to significantly influence health promotion behaviors, explaining 26.8% of the variance. In the third step, adding the mediating variable, psychological resilience, to the model explained an additional 29.7% of variance in health promotion behaviors. The regression coefficient for health literacy decreased from 0.743 in step two to 0.496 in step three, remaining statistically significant. The collinearity diagnosis indicates that the tolerance values for each model range from 0.232 to 0.805, and the variance inflation factors range from 1.233 to 4.313. Therefore, no multicollinearity exists among the independent variables. Preliminary statistical analysis indicates that psychological resilience partially mediates the relationship between health literacy and health promotion behaviors among older adult PCI patients (see Table 3).

**Table 3:** Multivariate stratified regression analysis of health promotion behaviors among older adult patients after PCI.

Variables	Model 1			Model 2			Model 3		
	*β*	*t*	*P*	*β*	*t*	*P*	*β*	*t*	*P*
Control variables									
Age	−0.116	−2.435	0.015	−0.089	−2.608	0.010	−0.072	−2.233	0.026
Average monthly family income	0.258	5.083	<0.001	0.095	2.516	0.012	0.079	2.200	0.029
Health literacy				0.743	16.623	<0.001	0.496	8.198	<0.001
Psychological resilience							0.317	5.745	<0.001
*F*		25.510**			70.483**			74.566**	
*R* ^2^		0.470			0.730			0.758	
*R*^2^ change		0.451			0.719			0.748	

**P < 0.001.

### 3.6 Testing the mediating effect of psychological resilience on the relationship between health literacy and health promotion behaviors in older adult patients after PCI

All variables were standardized. Using health literacy as the independent variable, health promotion behaviors as the dependent variable, psychological resilience as the mediating variable, and age and average monthly family income as control variables, mediation analysis was conducted using Model 4 in Process v4.1.

**Figure 2:**
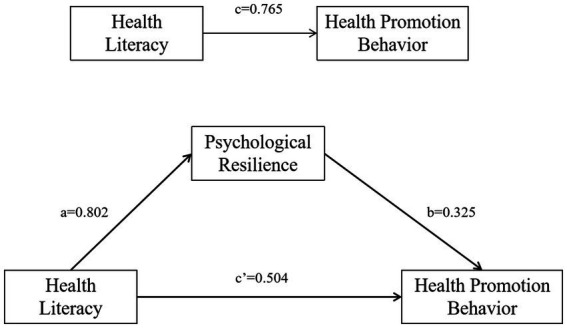
Schematic diagram of the mediating effect of psychological resilience between health literacy and health promotion behaviors.

The mediation analysis results revealed that health literacy positively predicted psychological resilience (*a* = 0.802, SE = 0.036, *p* < 0.001); health literacy positively predicted health promotion behaviors (*c*’ = 0.504, SE = 0.055, *p* < 0.001); psychological resilience positively predicted health promotion behaviors (*b* = 0.325, SE = 0.054, *p* < 0.001). The Bootstrap method revealed that psychological resilience partially mediated the positive relationship between health literacy and health promotion behaviors, with ab = 0.260, BootSE = 0.043, and a 95% CI of [0.175, 0.343]. The mediation effect accounted for 34.0% of the total effect (see Table 4 and Figure 2).

**Table 4:** Results of the mediating effect analysis.

Effects	Effect size	SE	95% CI
Total effect			
Health literacy → Health promotion behavior	0.765	0.036	(0.694，0.835)
Direct effect			
Health literacy → Health promotion behavior	0.504	0.055	(0.396，0.613)
Indirect effect			
Health literacy → Psychological resilience → Health promotion behavior	0.260	0.043	(0.175，0.343)

## 4 Discussion

This study investigated the level of health promotion behaviors among older adult patients in China following PCI procedures and employed a mediation model to validate the mediating role of psychological resilience in the relationship between health literacy and health promotion behaviors. Our findings reveal a significant positive correlation among health literacy, psychological resilience, and health promotion behaviors in older adult PCI patients. We further demonstrate that psychological resilience partially mediates the relationship between health literacy and health promotion behaviors. This study provides theoretical support for healthcare professionals to develop targeted interventions aimed at enhancing health literacy and psychological resilience in older adult patients with PCI, thereby further improving their health promotion behaviors.

### 4.1 Current status of health promotion behaviors among older adult patients after PCI

The study findings indicate that older adult patients after PCI scored (82.13 ± 11.78) points for health promotion behaviors, reflecting a moderate level. However, this score remains lower than the results reported by Chinese scholars Gong et al. (32) for patients with coronary heart disease (91.30 ± 11.83). Reasons for this discrepancy include: First, the study population comprised older adult PCI patients whose physiological functions and cognitive abilities gradually decline with age, manifesting as reduced memory and decreased mobility. This impairment affects their capacity to consistently adhere to medication regimens, attend regular follow-up appointments, and maintain moderate exercise routines (33). In contrast, the study by Gong et al. primarily involved middle-aged and young patients, a group with relatively better physiological conditions and greater physical strength and energy, providing more favorable objective conditions for implementing and maintaining healthy behaviors. Related studies also indicate that age is a significant factor contributing to variations in patient health behaviors. Older adult patients often suffer from multiple chronic diseases and symptoms such as fatigue and pain, further limiting their capacity for regular exercise and daily self-management (34). Additionally, the overall educational attainment of older adult patients in this study was generally low, which may have limited their ability to accurately comprehend health information and acquire knowledge for disease management. Lu et al. also demonstrated that insufficient health literacy directly impacts patients’ adherence to professional medical advice and the scientific rigor of their self-care behaviors (35). In contrast, the middle-aged and young patients studied by Gong et al. possessed relatively higher educational attainment, enabling them to more readily access health information through books, the internet, and other channels. This facilitated the development of scientifically grounded health knowledge, which in turn translated into proactive health promotion behaviors. Additionally, Gong et al.’s (32) study population comprised a broad range of patients with coronary artery disease, potentially including a significant number who had not undergone revascularization or were in a stable phase. In contrast, this study focused specifically on older adult patients following PCI. As an acute, invasive medical procedure, PCI may present patients with unique psychosocial challenges, such as persistent fear of restenosis, excessive preoccupation with cardiac function, and negative emotions associated with lifelong medication dependency. This intensified “patient role” and heightened disease uncertainty may create additional psychological barriers to maintaining proactive health behaviors, resulting in relatively lower behavioral scores. Furthermore, prior research indicates that older adult patients are more prone to negative emotions such as loneliness, anxiety, and depression due to factors like shifting social roles, declining physical function, and reduced social support. These psychological issues not only diminish their motivation to maintain healthy behaviors but may also further undermine treatment confidence and life enthusiasm, creating a vicious cycle between psychological state and behavior (36). Therefore, when assessing and improving health behaviors among older adult patients, it is essential to pay special attention to their unique psychological needs. The study results also reveal differences in health promotion behavior scores among older adult PCI patients across varying monthly household incomes, with patients from higher-income households exhibiting stronger health promotion behaviors—a finding consistent with previous research (37). This disparity stems from economic conditions serving as a crucial material foundation for sustaining health behaviors. Higher-income households can provide patients with better dietary options and effectively cover ongoing medical expenses such as regular follow-ups, out-of-pocket medications, and specialized rehabilitation equipment. These resources directly support the implementation and maintenance of health behaviors. Simultaneously, favorable economic conditions help alleviate the psychological burden caused by medical costs, enhancing patients’ confidence in treatment and motivation for recovery. This, in turn, further improves the adherence and sustainability of health behaviors (38).

### 4.2 Correlation analysis of health literacy, psychological resilience, and health promotion behaviors

The results of this study indicate a significant positive correlation between health literacy and health promotion behaviors (*r* = 0.840, *p* < 0.01), suggesting that older adult patients undergoing PCI exhibit higher levels of health promotion behaviors as their health literacy increases. This finding aligns with previous research (39). On one hand, higher health literacy provides the knowledge foundation for patients to correctly understand and implement health behaviors. Patients with high health literacy possess a deeper understanding of disease knowledge, treatment modalities, and key postoperative rehabilitation points. They clearly recognize the importance of health-promoting behaviors—such as taking medication on time, maintaining a balanced diet, and engaging in moderate exercise—for recovery. This facilitates the translation of abstract medical advice into concrete, actionable daily practices (35). On the other hand, patients with strong health literacy not only possess more solid knowledge reserves but also demonstrate superior information acquisition and discernment abilities. They proactively utilize multiple channels—such as medical guidance, science-based materials, or reliable online resources—to address challenges encountered during recovery. They effectively filter out false health information, make healthier decisions, and continuously refine their self-management strategies (40). Furthermore, research indicates that when patients successfully manage health issues through their knowledge base, their confidence in overcoming illness and sense of self-efficacy significantly increase. This positive psychological feedback transforms into an internal driving force for maintaining healthy behaviors, enabling them to sustain good compliance throughout the long-term rehabilitation process (41).

The results of this study indicate a significant positive correlation between health literacy and psychological resilience (*r* = 0.844, *p* < 0.01), suggesting that older adult patients undergoing PCI exhibit stronger psychological resilience with higher health literacy—a finding consistent with previous research (42). Research indicates that older adult patients often experience negative emotions such as loneliness, anxiety, and depression due to factors including physical decline, shrinking social circles, and multiple pressures stemming from illness. These psychological issues not only directly impair their mental health but also weaken their willingness and ability to manage their health (43). When older adult PCI patients possess strong health literacy, they gain a more comprehensive and in-depth understanding of disease knowledge, treatment methods, and rehabilitation precautions. This enables them to confront illness with a more positive and rational mindset, thereby enhancing psychological resilience. As a result, they can maintain optimism when facing postoperative physical discomfort and lifestyle changes, better adapt to new living conditions, and promote comprehensive physical and mental recovery (44).

The results of this study indicate that psychological resilience exhibits a significant positive correlation with health-promoting behaviors (*r* = 0.811, *p* < 0.01). This suggests that older adult patients undergoing PCI demonstrate stronger health-promoting behaviors as their psychological resilience increases, consistent with previous research findings (45). As a positive individual psychological resource, psychological resilience serves as a core capacity for coping with disease-related stress and challenges (20). Research indicates that older adult patients with robust psychological resilience exhibit superior emotional regulation and stress-coping abilities. When confronting difficulties and setbacks during rehabilitation, they recover more quickly from negative emotions and maintain a positive attitude toward treatment. This psychological advantage not only facilitates consistent treatment adherence but also encourages patients to proactively adopt and sustain healthy lifestyles, thereby significantly enhancing their overall health promotion behaviors (46). Furthermore, research by Wang et al. suggests that patients with higher psychological resilience tend to exhibit stronger problem-solving abilities. This enables them to develop more effective rehabilitation plans and proactively seek social support and professional assistance when encountering barriers to implementing health behaviors. Consequently, a virtuous cycle emerges where psychological resources and health behaviors mutually reinforce each other (47).

### 4.3 Mediating role of psychological resilience between health literacy and health promotion behaviors

The findings reveal that psychological resilience mediates the relationship between health literacy and health promotion behaviors. Specifically, health literacy not only directly influences health promotion behaviors among older adult PCI postoperative patients but also indirectly affects these behaviors through the mediating effect of psychological resilience, accounting for 34.0% of the total effect. Specifically, health literacy—as the key ability for individuals to access, understand, and apply health information—effectively enhances patients’ disease cognition and self-management confidence, thereby positively influencing their psychological resilience (13). Psychological resilience, as the core psychological resource for coping with stress and adversity, can significantly improve health promotion behaviors by strengthening patients’ adaptability and persistence. Research indicates that compared to patients with lower psychological resilience, those with higher psychological resilience are better equipped to effectively cope with challenges during the rehabilitation process and proactively adjust unhealthy lifestyles, thereby forming and maintaining positive health behaviors (47). According to the Health Promotion Model, individual traits and experiences, along with behavior-specific cognition and emotions, collectively influence health behavior decisions. This theory emphasizes the pivotal role of cognitive psychological factors in behavioral change (23). On one hand, health literacy, as a crucial cognitive factor, enables patients with higher health literacy to accurately comprehend health information and translate it into practical actions, thereby promoting their health behaviors. On the other hand, higher health literacy enhances patients’ ability to adapt to stress and regulate their emotions, thereby increasing their psychological resilience and indirectly improving their health behaviors (48). Chen et al. also demonstrated that higher health literacy strengthens patients’ self-efficacy and confidence in disease management, leading to more proactive implementation of self-management-related health behaviors (42).

### 4.4 Implications for practice

This study confirms the partial mediating role of psychological resilience in the relationship between health literacy and health promotion behaviors among older adult PCI patients in China. This finding provides a critical, integrated new perspective for improving health management practices in this population.

First, for clinical healthcare providers, assessing and cultivating psychological resilience must be regarded as a core component of patient evaluation and guidance, equally important to health education. This implies that during clinical communication, beyond inquiring “Do you know what to do?,” greater attention should be paid to “What psychological or emotional difficulties have you encountered in implementation?” and providing support accordingly.

Second, for health educators and rehabilitation counselors, educational content should extend beyond knowledge transmission to organically integrate psychological empowerment components. For instance, when discussing medication adherence, simultaneously teach patients techniques to manage anxiety after missed doses; when designing exercise plans, help patients set achievable small goals to enhance self-efficacy. This fundamentally embeds psychological resilience cultivation strategies within the process of improving health literacy.

Finally, for public health and rehabilitation program administrators, resource allocation and program design must recognize that supporting older adult PCI patients involves two critical phases: an initial “empowerment” stage focused on health literacy (providing clear, accessible information), followed by a later “transformation” stage centered on psychological resilience (delivering sustained psychosocial support to overcome behavioral barriers). When evaluating program effectiveness, improvements in both health literacy and psychological resilience should be incorporated as process indicators.

### 4.5 Specific recommendations and future intervention directions

Based on the insights above, we further propose the following more actionable recommendations to provide reference for developing comprehensive intervention measures in the future:

(1) Precision strategies for enhancing health literacy among older adult patients: Given the information reception characteristics of older adult, low-literacy, and low-income patients, multi-tiered health education interventions should be implemented. Content design should prioritize developing rehabilitation guidance materials featuring illustrations supplemented by text, focusing on practical skills such as medication identification and administration, symptom recognition, and dietary planning. In implementation, community health centers should establish regular follow-up systems to reinforce key information delivery through face-to-face demonstrations and group lectures. Simultaneously, encourage family members to participate in the health education process, leveraging their supportive role in reinforcing information and monitoring behavior. For low-income groups, healthcare institutions are advised to provide free basic rehabilitation kits to help translate health knowledge into concrete actions.

(2) Supportive interventions to enhance psychological resilience: To address common anxiety, depression, and uncertainty about illness among older adult patients, a professional psychosocial support network should be established. At the institutional level, dedicated psychological clinics for post-PCI patients should be established, where healthcare professionals regularly assess patients’ psychological states and provide cognitive-behavioral therapy guidance to help them develop a positive disease cognition. At the community level, organize patient support groups where patients share their successful recovery stories to bolster hope and self-efficacy. Simultaneously, guide family members in recognizing patients’ psychological needs, offering emotional support and companionship to alleviate loneliness and psychological burdens.

(3) Establish a comprehensive support system for economically disadvantaged groups: For patients from households with lower monthly incomes, it is recommended to alleviate their financial burdens through multiple channels. Promote the optimization of medical insurance policies by including key post-PCI follow-up examinations and essential rehabilitation equipment within the scope of reimbursement. Medical institutions may establish “rehabilitation assistance funds” to subsidize out-of-pocket medication costs for financially disadvantaged patients. Concurrently, community health centers should provide complimentary basic rehabilitation assessments and guidance, while organizing group rehabilitation activities to reduce the economic burden for individual patients seeking standalone rehabilitation training. Encourage community pharmacies to regularly offer discounted medications for chronic diseases, employing multiple approaches to alleviate patients’ financial strain and create the necessary conditions for sustaining healthy behaviors.

## 5 Strengths and limitations

This study possesses certain theoretical and practical advantages. First, at the theoretical level, grounded in the health promotion model, this research empirically tested and confirmed the mediating role of psychological resilience in the relationship between health literacy and health promotion behaviors among older adult Chinese patients after PCI. This finding not only addresses previous research gaps in exploring the intrinsic mechanisms among these three factors but also integrates health literacy—a cognitive factor—with psychological resilience—a psychodynamic factor—into a coherent theoretical framework. It provides a more nuanced “cognitive-psychological” dual-pathway explanation for understanding the formation mechanisms of health behaviors in this population, enriching the application of health behavior theory within this specific group. Second, methodologically, this study employed validated standardized scales to measure core variables and utilized the Bootstrap method to test mediating effects, enhancing the statistical power and reliability of the mediation model analysis. Finally, despite the single source of the sample, this study focused on a highly homogeneous and vulnerable clinical group (older adult Chinese patients after PCI), clearly revealing the intrinsic relationships among variables. This provides important preliminary evidence and mechanistic hypotheses for subsequent research in broader populations.

This study also has certain limitations. First, due to limitations in human, material, and financial resources, this study exclusively surveyed older adult patients who underwent percutaneous coronary intervention (PCI) in the cardiovascular department of a Grade A tertiary hospital in Sichuan Province, China. Given variations in medical resources, economic development levels, cultural practices, and patient characteristics across different regions of China, the findings primarily reflect conditions within this specific healthcare setting. Caution is advised when extrapolating these results to other regions nationwide or to healthcare institutions of different tiers. Future research should conduct multicenter, large-sample surveys encompassing diverse geographic regions and hospitals of varying levels to validate and expand upon the findings of this study. Second, as a cross-sectional study, this research cannot establish causal relationships and fails to assess longitudinal trajectories of health literacy, psychological resilience, and health promotion behaviors among older adult PCI patients. Future longitudinal studies are needed to examine trends in these three domains. Furthermore, this study primarily relies on patient self-reports, which may be subject to social desirability bias and recall bias. Such homogenous biases could generate spurious correlations between variables, potentially amplifying the observed strength of associations between health literacy, psychological resilience, and health-promoting behaviors. Although we endeavored to minimize social desirability effects during the survey by emphasizing anonymity, confidentiality, and the absence of right or wrong answers, this methodological limitation remains unavoidable. Future research should employ multi-method, multi-source data validation incorporating objective metrics and third-party assessments from healthcare providers or family members to more accurately capture genuine relationships among variables. Finally, while the scales used in this study possess some validity evidence among similar populations in China, their measurement equivalence and construct validity within the specific subgroup of “older adult PCI postoperative patients” remain insufficiently validated. Future research may conduct more in-depth validity testing of the scale for this group or develop more context-specific measurement tools.

## 6 Conclusion

In summary, health promotion behaviors among older adult patients after PCI in China are at a moderate level. Health literacy not only directly influences patients’ health promotion behaviors but also indirectly affects them through psychological resilience. This finding confirms that psychological resilience serves as a crucial psychological bridge connecting cognitive resources (health literacy) with actual actions (health behaviors). It not only provides an important dual-pathway explanatory model—cognitive and psychological—for understanding the complex process of health behavior formation in this population, but also suggests that future clinical care and cardiac rehabilitation practices should shift from merely providing information to adopting an empowerment model that integrates psychosocial support and aims to enhance patients’ intrinsic resilience. Future longitudinal studies or randomized controlled trials are recommended to confirm the causal relationship among health literacy, psychological resilience, and health promotion behaviors, while dynamically tracking their trajectory. Furthermore, based on the revealed mediating pathway in this study, comprehensive intervention programs designed to simultaneously enhance health literacy and psychological resilience should be developed and tested for efficacy. This will provide stronger evidence for constructing integrated cardiac rehabilitation strategies grounded in evidence and centered on patients’ holistic health.

## Data Availability

The original contributions presented in the study are included in the article/supplementary material, further inquiries can be directed to the corresponding authors.
